# Supplementary material for: Improving the Performance of Polymer Solar Cells with Benzo[ghi]perylenetriimide-Based Small-Molecules as Interfacial Layers
Source: Polymers (Basel). 2022 Oct 21;14(20):4466. doi: 10.3390/polym14204466 (PMC9607574; doi:10.3390/polym14204466)
Supplement: Supplementary file 1 [file polymers-14-04466-s001.zip › polymers-1943334-supplementary.pdf]

Supplementary Materials

Experimental Section

N^1,N^2-Bis(3-pentyl)-N^3-(1-aminopropyl)benzo[ghi]perylene-2,3,8,9,11,12-hexacarboxylictriimide (BPTI-C_3NH_2)

A solution of the anhydride precursor-(a) [29] (600 mg, 0.960 mmol) and 1,3-diaminopropane (0.800 mL, 9.62 mmol) in THF (80 mL) and toluene (120 mL) was heated at 80 °C for 4 h under an Ar atmosphere. The suspension was cooled and then the mixture was concentrated under reduced pressure. The suspended material was filtered off and washed with MeOH. The crude product was dissolved in CF and purified through column chromatography (SiO_2; $CHCl_3$/acetone, 95:5) to afford BPTI-C_3NH_2 (352 mg, 53%). ^{1}H NMR (400 MHz, $CDCl_3$) (as shown in Figure S1): δ (ppm) = 9.77 (s, 2H), 8.87 (d, *J* = 8.0 Hz, 2H), 8.77 (d, *J* = 8.0 Hz, 2H), 5.18–5.14 (m, 2H), 3.97 (t, J = 4.0 Hz, 2H), 2.40 (m, 4H), 2.16–2.06 (m, 4H), 1.94–1.85 (m, 2H), 1.54–1.49 (m, 2H), 1.11–1.06 (m, 14H). ^{13}C NMR (400 MHz, $CDCl_3$) (as shown in Figure S1): δ (ppm) = 167.79, 132.26, 127.1, 126.87, 126.36, 123.8, 123.35, 122.35, 58.11, 38.60, 30.83, 25.32, 20.44, 13.90, 11.85. MALDI-TOF MS: calcd, *m*/*z* 680.263; found, 680.291 $[M]^+$, 681.302 $[M + H]^+$

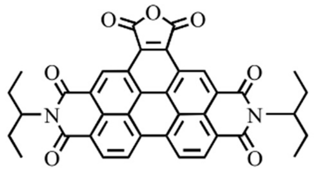

BPTI compound (a).

Ammonium Iodide Salt BPTI-C_3NH_3I

Hydriodic acid (57 wt. % in H_2O, 66.0 mL) was added to a solution of BPTI-C_3NH_2 (300 mg, 0.440 mmol) in CF (120 mL) and EtOH (10 mL) at room temperature. The solution was evaporated to dryness using a rotary evaporator. Et_2O (50 mL) was added to the residue. The precipitate was filtered off and washed with Et_2O. Drying under vacuum afforded BPTI-C_3NH_3I as an orange solid (340 mg, 95%). MALDI-TOF MS: calcd, *m*/*z* 681.271; found, 681.303 $[M]^+$.

N^1,N^2-Bis(3-pentyl)-N^3-(3-dimethylaminopropyl)benzo[ghi]perylene-2,3,8,9,11,12-hexacarboxylictriimide (BPTI-C_3DMA)

A mixture of the anhydride precursor-(a) (700 mg, 1.90 mmol) and 3-dimethylaminopropylamine (0.400 mL, 3.37 mmol) in toluene (100 mL) and N, N-Dimethylformamide (DMF) (30 mL) was heated at 110 °C for 6 h under an N_2 atmosphere. After evaporation of the solvent, MeOH (50 mL) was added to the residue. The precipitate was filtered off and washed with MeOH. The crude product was dissolved in CF and purified through column chromatography (SiO_2; CF/MeOH, 50:1) to afford BPTI-C_3DMA as a yellow solid (650 mg, 81%). ^{1}H NMR (400 MHz, $CDCl_3$) (as shown in Figure S2): δ (ppm) = 9.53 (s, 2H), 8.77 (d, *J* = 8.0 Hz, 2H), 8.51 (d, *J* = 8.0 Hz, 2H), 5.17–5.10 (m, 2H), 3.99 (t, *J* = 4.0 Hz, 2H), 2.57 (t, *J* = 4.0 Hz, 2H), 2.45–2.39 (m, 2H), 2.37–2.34 (m, 6H), 2.19–2.08 (m, 8H), 1.12 (t, *J* = 4.0 Hz, 12H). ^{13}C NMR (400 MHz, $CDCl_3$) (as shown in Figure S2): δ (ppm) = 167.15, 131.37, 129.76, 127.99, 126.46, 126.17, 125.39, 122.96, 122.61, 121.49, 58.37, 57.04, 45.28, 36.94, 25.33, 26.36, 24.97, 11.59. MALDI-TOF MS: calcd, *m*/*z* 708.295; found, 709.307 $[M + H]^+$.

Device Fabrication

The following steps were applied to prepare BHJ devices with the structure ITO/ZnO/IFL/active layer/MoO_3/Ag. (1) Preparation of ITO substrate: An ITO glass substrate (Japan Sanyo; 8 Ω/□) was patterned through a photolithographic process and then

subjected to washing (detergent), ultrasonic treatment [acetone, water, and isopropyl alcohol (IPA)], drying (140 °C, 10 min), and further drying and cleaning with O_2 plasma (5 min). (2) Preparation of ETL: Zinc acetate, ethylene glycol methyl ether, and ethanolamine were used to prepare the ZnO layer through a sol–gel method, stirring at 300 rpm for 72 h and then passing through a 0.45-μm polytetrafluoroethylene (PTFE) filter and spin-coating onto the ITO substrate at 3000 rpm in air. The obtained wet film material was dried at 170 °C for 20 min (thickness: ca. 40 nm). (3) Preparation of IFL: Solutions of BPTI-C_3DMA, BPTI-C_3NH_3I, and BPTI-C_3NH_2 were prepared at various concentrations (0.5, 1, and 1.5 mg mL^{-1}), and then they were spin-coated (2000 rpm, 30 s) on top of the ZnO. CF and MeOH were then applied to perform SA. (4) Preparation of active layers: $PC_{71}BM$ and PTB7-Th (1:1.5) were stirred overnight in chlorobenzene (CB) in a glove box and then spin-coated (2500 rpm, 30 s) onto the top surface of the IFL (thickness: ca. 100 nm). (5) Preparation of HTL: MoO_3 (3 nm) and a layer of Ag (thickness: 100 nm) were deposited onto the active layer under a pressure of less than 10^{-6} torr. The effective area of this device was 0.1 cm^2. Ten devices were used to measure the average values of performance characteristics, presented as the final data.

Characterization and Measurement

^{1}H and ^{13}C NMR spectroscopy were performed using a Bruker AMX 400 spectrometer. Mass spectrometry (MALDI-TOF-MS) was performed using Bruker Daltonics and Autoflex Speed mass spectrometers; a 2-[(2E)-3-(4-tert-Butylphenyl)-2-methylprop-2-enylidene] malononitrile (DCTB) matrix was used for the MALDI-TOF-MS measurements. UV–Vis spectra were recorded using a Jasco V-650 UV–Vis spectrophotometer with CB as the solvent. The surface morphologies of the blend films were analyzed using a Bruker Dimension Edge atomic force microscope, operated in tapping mode at ambient temperature. The ***OPV***performance was measured in an Ar-filled glove box. A computer-controlled Keithley 2400 light source measurement unit and a Peccell solar simulator were used to measure the *J*–*V* characteristics of the OPV devices under AM 1.5G illumination (1000 W m^{-2}). The illumination intensity was calibrated using a standard Si reference cell and a KG-5 filter. EQE spectra were measured using an Enlitech QE-R spectral response measurement system to calibrate the current densities of the devices. The exciton dissociation probability [*P*(E,T)] was calculated using the equations[59]

$$J_{ph} = J_{light} - J_{dark} \tag{1}$$

$$V_{eff} = V_0 - V_a \tag{2}$$

$$J_{sat} = q\, G_{max}\, L \tag{3}$$

$$J_{ph} = q\, G_{max}\, P(E,T)\, L \tag{4}$$

where J_{dark} is the dark current density; J_{light} is the photocurrent density; V_0 is the voltage at a value of J_{ph} of 0 mA cm^{-2}; V_a is the applied voltage; *q* is elementary charge; *L* is the thickness of the active layer; and G_{max} is the maximum number of absorbed photons.

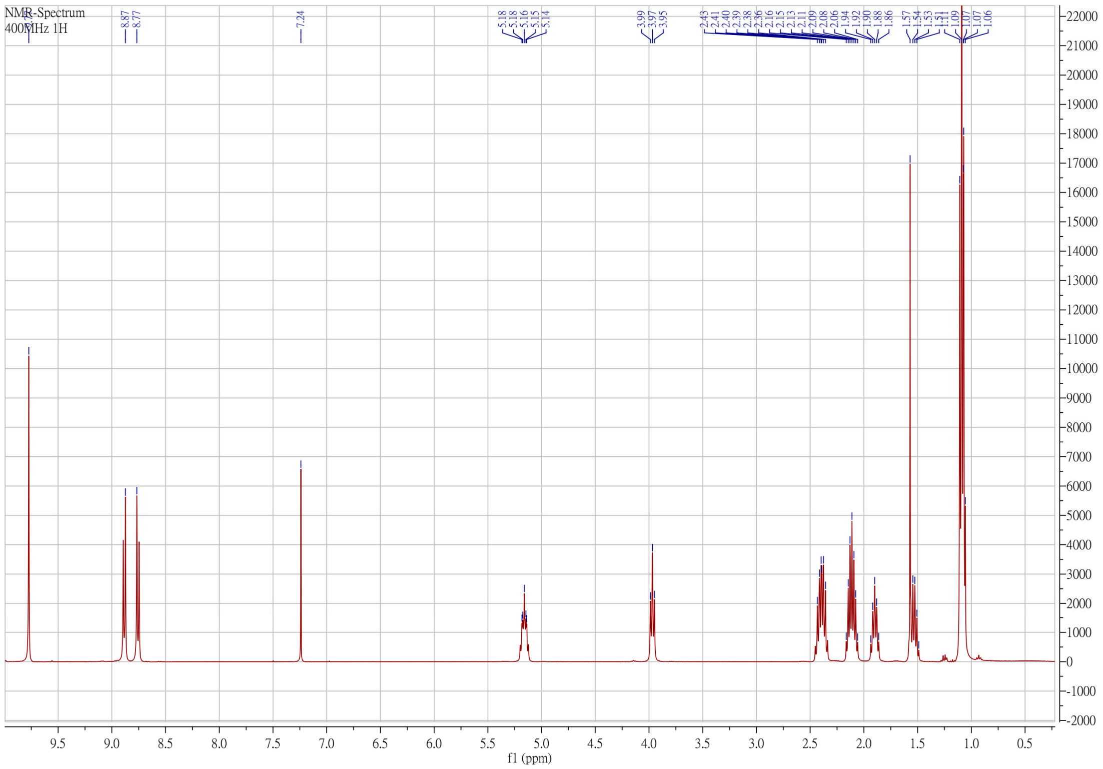

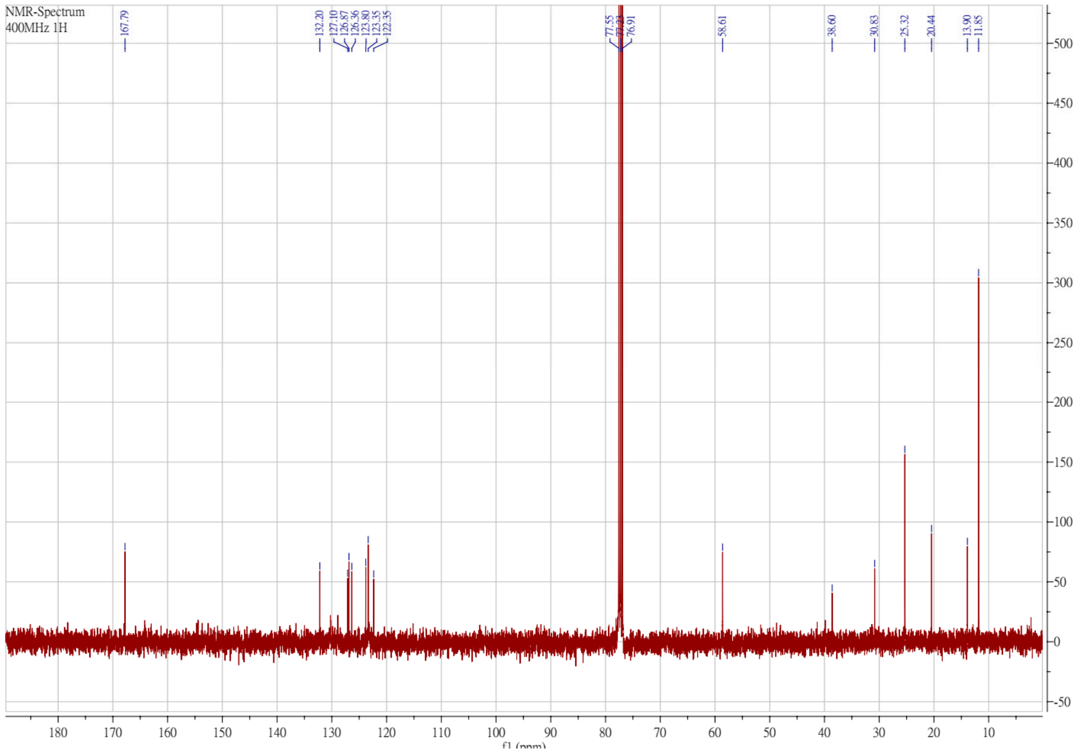

Figure S1. (top) ^{1}H and (bottom) ^{13}C NMR spectra of BPTI-C_3NH_2 in $CDCl_3$.

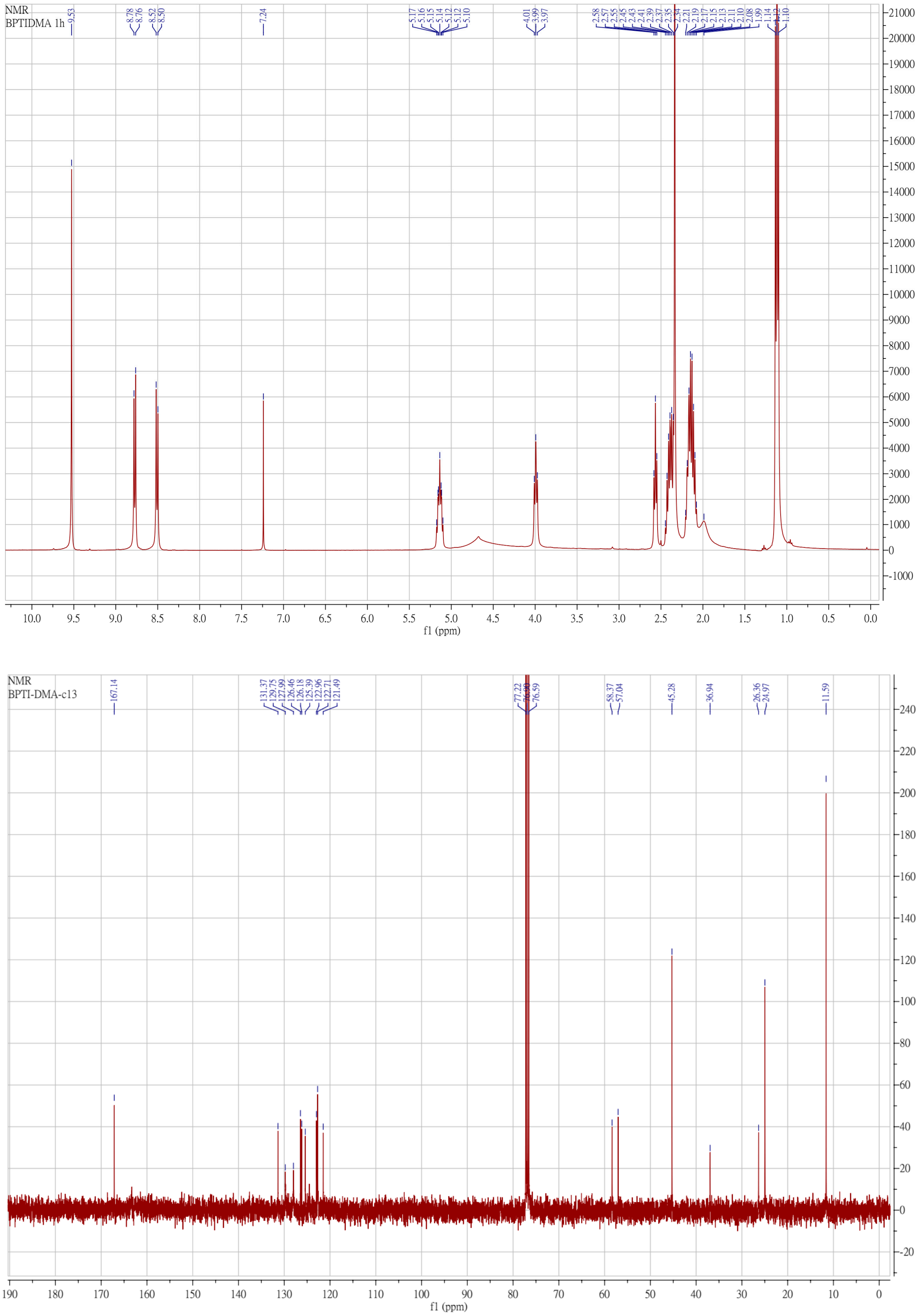

Figure S2. (top) ^{1}H and (bottom) ^{13}C NMR spectra of BPTI-C_3DMA in $CDCl_3$.

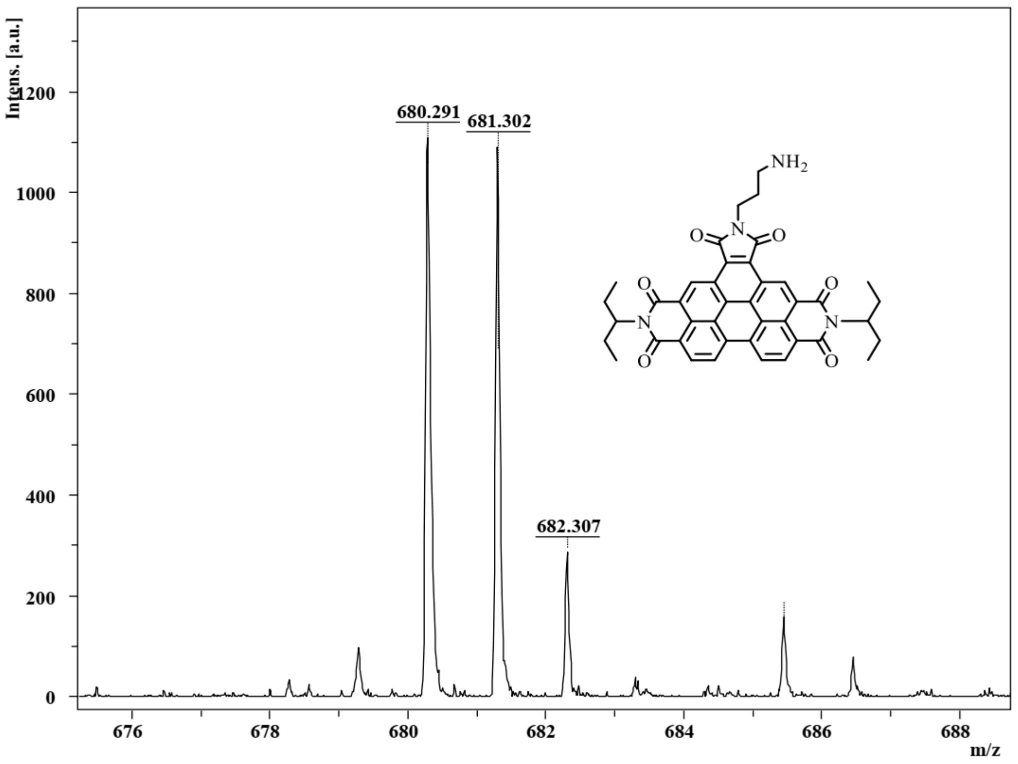

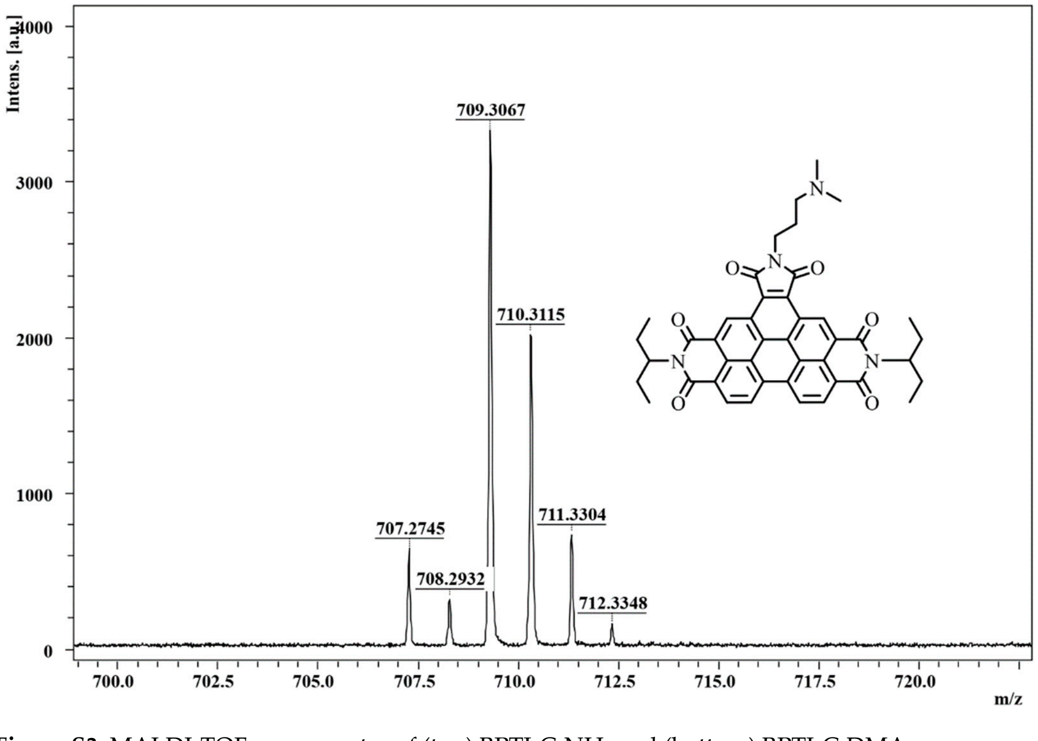

Figure S3. MALDI-TOF mass spectra of (top) BPTI-C_3NH_2 and (bottom) BPTI-C_3DMA.

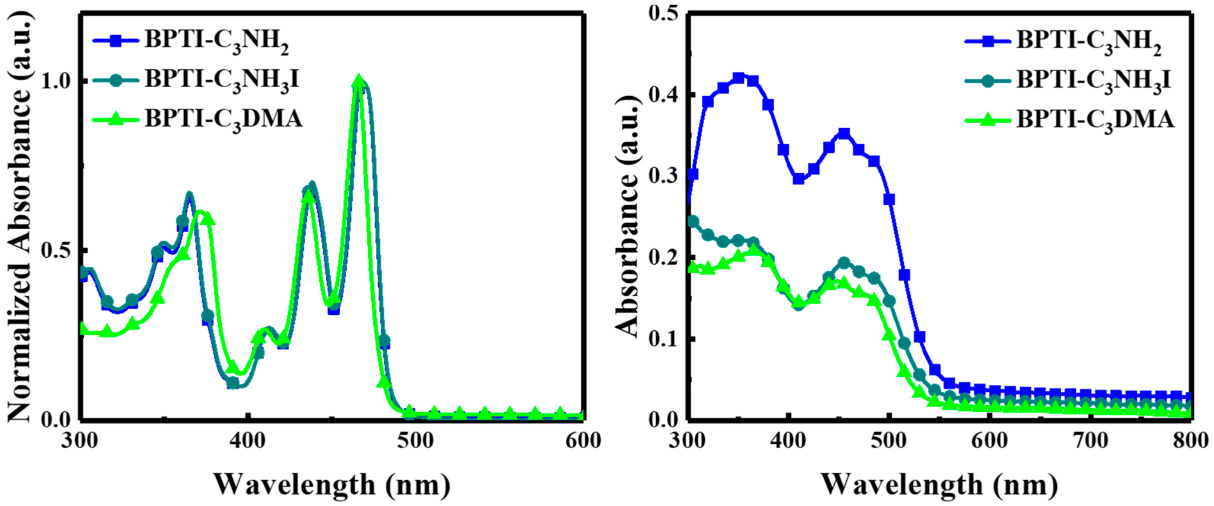

(**a**) (**b**)

Figure S4. UV–Vis absorption spectra of the BPTI derivatives (**a**) as solutions in $CHCl_3$ and (**b**) in the form of glass/BPTI substrates.

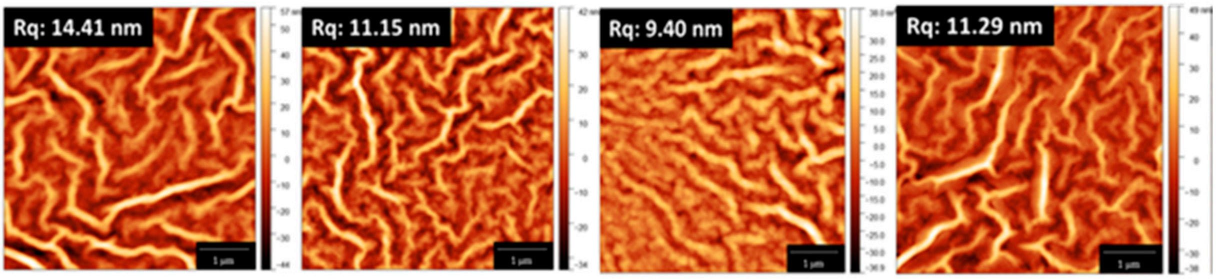

(**a**) (**b**) (**c**) (**d**)

Figure S5. Tapping-mode AFM images of the (**a**) ZnO, (**b**) ZnO/BPTI-C_3NH_2, (**c**) ZnO/BPTI-C_3NH_3I, and (**d**) ZnO/BPTI-C_3DMA samples (5 µm × 5 µm).

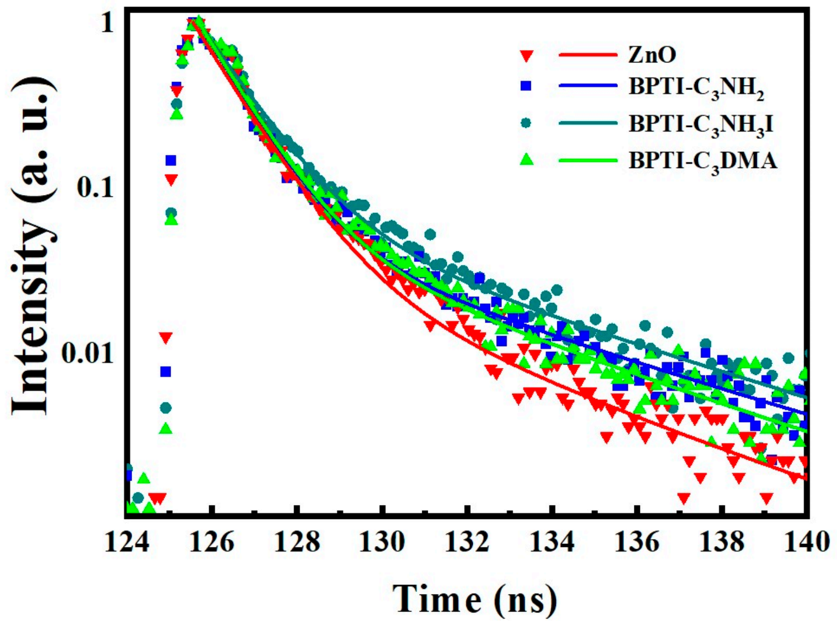

Figure S6. TRPL spectra of blend films prepared with and without IFLs.

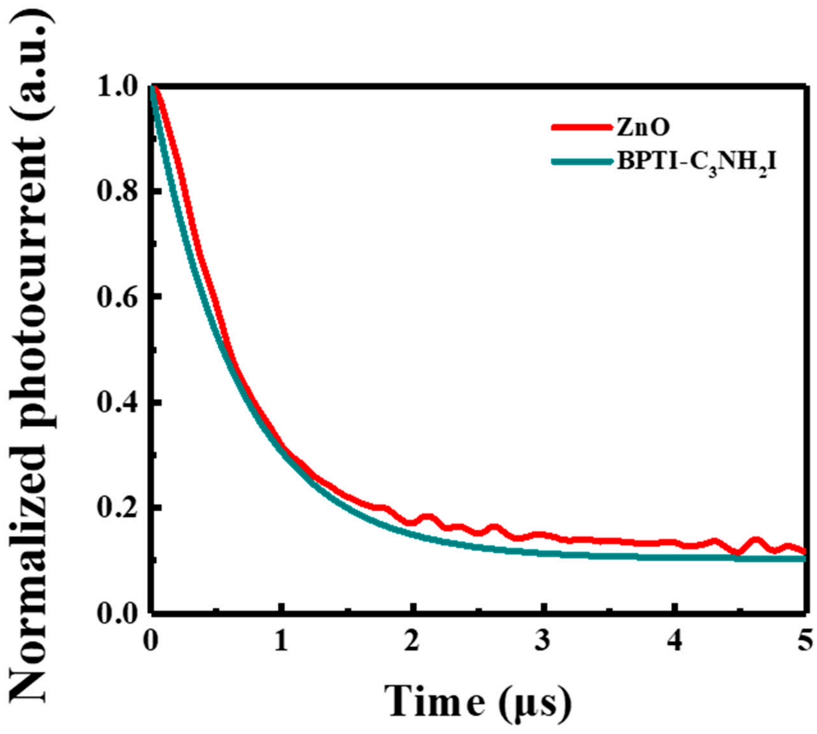

Figure S7. Normalized TPC data for the control and BPTI-C_3NH_3I-modified devices.

Table S1. Contact angles and surface energies of SA-treated IFLs.

**ZnO/BPTI-C_3NH_3I**	θ_{water} (°)	θ_{DIM} (°)	γ_{polar} (mN m^{-1})	$\gamma_{dispersive}$ (mN m^{-1})	γ_{total} (mN m^{-1})
As-coated	60.60	26.95	16.36	45.57	61.93
CF_SA	66.34	21.49	13.34	47.38	60.73
MeOH_SA	55.00	32.65	19.55	43.36	62.91

Table S2. Carrier lifetime parameters of the blend films.

	τ_1 **(ns)**	τ_2 **(ns)**	τ_{avg} **(ns)**
ZnO	5.100	0.9512	1.202
BPTI-C_3NH_2	4.158	1.0134	1.743
BPTI-C_3NH_3I	5.076	1.0803	1.997
BPTI-C_3DMA	4.628	0.972	1.648
